# Supplementary material for: Morphoregulatory ADD3 underlies glioblastoma growth and formation of tumor–tumor connections
Source: Life Sci Alliance. 2024 Nov 26;8(2):e202402823. doi: 10.26508/lsa.202402823 (PMC11599137; doi:10.26508/lsa.202402823)
Supplement: Supplementary file 6 [file LSA-2024-02823_TableS1.docx]

**Table S1. Reagents and Tools table**

| **Reagent/Resource** | **Reference or Source** | **Identifier or Catalog Number** |
| --- | --- | --- |
| **Experimental Models** |  |  |
| Onda-11 cell line (human) | Tebu-Bio | JCRB1605 |
| U-87 MG cell line (human) | ATCC | HTB-14™ |
| H4 cell line (human) | ATCC | HTB-148™ |
| **Recombinant DNA** |  |  |
| pCAG | (Kalebic et al., 2019) | N/A |
| pCAG-GFP | (Kalebic et al., 2019) | N/A |
| pCAG-ADD3 | This paper | N/A |
| pCas9-LacZ | This paper | N/A |
| pCas9-gADD3_ex4 | This paper | N/A |
| pCas9-gADD3_ex6 | This paper | N/A |
| **Antibodies** |  |  |
| Mouse anti-Nestin polyclonal antibody (1:500 IF cells and tissue) | Invitrogen | MA1-110 |
| Rabbit anti-OCT4 polyclonal antibody (1:200 IF cells and tissue) | Abcam | Ab19857 |
| Rabbit anti-ADD3 polyclonal antibody (1:500 IF cells; 1:1000 WB) | Sigma-Aldrich | HPA035696 |
| Rabbit anti Cleaved-Caspase3 polyclonal antibody (1:200 IF cells) | Abcam | ab2302 |
| Rabbit anti-GFAP polyclonal antibody (1:500 IF cells) | DAKO | Z0334 |
| Rat anti-CD44 monoclonal antibody (1:500 IF cells) | Invitrogen | MA4400 |
| Mouse anti-A2B5 monoclonal antibody (1:200 IF cells) | Sigma-Aldrich | MAB312 |
| Mouse anti-p(ser55) Vimentin monoclonal antibody (1:500 IF cells) | MBL | D076-3 |
| Rabbit anti-Ki-67 monoclonal antibody (1:500 IF cells) | Cell Signalling | 9129 |
| Mouse anti-L1CAM monoclonal antibody (1:200 IF cells) | Abcam | Ab24345 |
| Mouse anti-AlphaTubulin monoclonal antibody (1:500 IF cells) | Millipore-Sigma | T5168 |
| Mouse anti-Actin monoclonal antibody (1:20.000, WB) | Millipore-Sigma | MAB15 |
| Donkey anti-Rabbit Alexa Fluor PLUS 488 (1:500 IF cells) | Invitrogen | A32790 |
| Donkey anti-Rabbit Alexa Fluor PLUS 555 (1:500 IF cells and tissue) | Invitrogen | A32794 |
| Donkey anti-Rabbit Alexa Fluor PLUS 647 (1:500 IF cells) | Invitrogen | A32790 |
| Donkey anti-Mouse Alexa Fluor PLUS 488 (1:500 IF cells and tissue) | Invitrogen | A32766 |
| Donkey anti-Mouse Alexa Fluor PLUS 555 (1:500 IF cells) | Invitrogen | A32773 |
| Donkey anti-Mouse Alexa Fluor PLUS 647 (1:500 IF cells) | Invitrogen | A32787 |
| Donkey anti-Rat Alexa Fluor PLUS 555 (1:500 IF cells) | Invitrogen | A48270 |
| Donkey anti-Rat Alexa Fluor PLUS 647 (1:500 IF cells) | Invitrogen | A48272 |
| Goat anti-Rabbit-HRP conjugated (1:5000, WB) | BioRad | 1706515 |
| Goat anti-Mouse-HRP conjugated (1:5000, WB) | BioRad | 1706516 |
| **Oligonucleotides and other sequence-based reagents** |  |  |
| ADD3 Exon 4 gRNA | This study | GTCTACAAGTCTGTACAGGCTGG |
| ADD3 Exon 6 gRNA | This study | GTTGAATAGATTGCAGCATGGGG |
| LacZ gRNA | (Kalebic et al., 2016) | TGCGAATACGCCCACGCGAT |
| **Chemicals, Enzymes and other reagents** |  |  |
| Human EGF Recombinant Protein | PeproTech | 100-15 |
| Human FGF-basic (FGF-2/bFGF) (154 aa) | PeproTech | 100-18B |
| Cytochalasin D | Sigma-Aldrich | C8273 |
| Temozolomide | Sigma-Aldrich | T2557 |
| DAPI | Invitrogen | 62248 |
| Alexa Fluor 647 Phalloidin | Invitrogen | A22287 |
| Matrigel | Corning | FLC354234 |
| **Software and Data** |  |  |
| Fiji ImageJ | v2.9.0 | [Fiji (imagej.net)](https://imagej.net/software/fiji/) |
| GraphPad Prism | v9.1.2 | [Home - GraphPad](https://www.graphpad.com/) |
| CellPose | v2.0 | [cellpose](https://www.cellpose.org/) |
| Python | v3.12.1 | <https://www.python.org/> |
| Zeiss Zen Blue | v3.7 | [ZEISS ZEN Microscopy Software](https://www.zeiss.com/microscopy/en/products/software/zeiss-zen.html) |
| nf-core/rnaseq | v3.13.2 | <https://nf-co.re/rnaseq/3.13.2> |
| VennDiagram | v1.7.3 | [https://cran-r--project-org.translate.goog/web/packages/VennDiagram/](https://cran-r--project-org.translate.goog/web/packages/VennDiagram/index.html?_x_tr_sl=en&_x_tr_tl=it&_x_tr_hl=it&_x_tr_pto=tc) |
| ggplot2 | v3.4.2 | <https://cran.r-project.org/src/contrib/Archive/ggplot2/> |
| CoRe | v1.0.0 | [https://rdrr-io.translate.goog/github/DepMap-Analytics/CoRe/](https://rdrr-io.translate.goog/github/DepMap-Analytics/CoRe/?_x_tr_sl=en&_x_tr_tl=it&_x_tr_hl=it&_x_tr_pto=tc) |
| DESeq2 | v1.34.0 | <https://bioconductor.org/packages/3.18/bioc/src/contrib/Archive/DESeq2/> |
| pheatmap | v1.0.12 | <https://rdrr.io/cran/pheatmap/> |
| Cancer Cell Line Encyclopedia (CCLE) expression dataset | v22Q2 | <https://depmap.org/portal/download/all/> |
| Cell Model Passport (CMP) | v20210611 | [https://cog.sanger.ac.uk/cmp/download/](https://cog.sanger.ac.uk/cmp/download/model_list_20210611.csv) |
| Project Score Sanger-Broad combined dataset | v1-v20Q2 | [https://cog.sanger.ac.uk/cmp/download/](https://cog.sanger.ac.uk/cmp/download/Project_score_combined_Sanger_v1_Broad_20Q2_20210311.zip) |
| **Other** |  |  |
| Lipofectamine™ Stem Transfection Reagent | Invitrogen | STEM008 |
| Lonza™ P3 Primary Cell 4D-Nucleofector™ X Kit | Lonza | V4XP-3024 |
| Fugene HD | Promega | E2311 |
| Click-iT™ Plus EdU Alexa Fluor™ 647 | Invitrogen | C10340 |
| QIAquick PCR Purification Kit | QIAGEN | 28104 |
| EndoFree Plasmid Maxi kit | QIAGEN | 12362 |
| QIAquick gel extraction kit | QIAGEN | 28704 |
| SMART-Seq® v4 Ultra® Low Input RNA Kit for Sequencing | Takara Bio | 634893 |
| MethoCult™ Methylcellulose-Based Media | Stem Cell Technologies | SF H4236 |
